# Supplementary material for: Detection of PCR chimeras in adaptive immune receptor repertoire sequences
Source: Bioinformatics. 2025 Oct 22;41(11):btaf576. doi: 10.1093/bioinformatics/btaf576 (PMC12619642; doi:10.1093/bioinformatics/btaf576)
Supplement: btaf576_Supplementary_Data [file btaf576_supplementary_data.zip › Chernyshev_etal_2025_Supplementary_Figures.pdf]

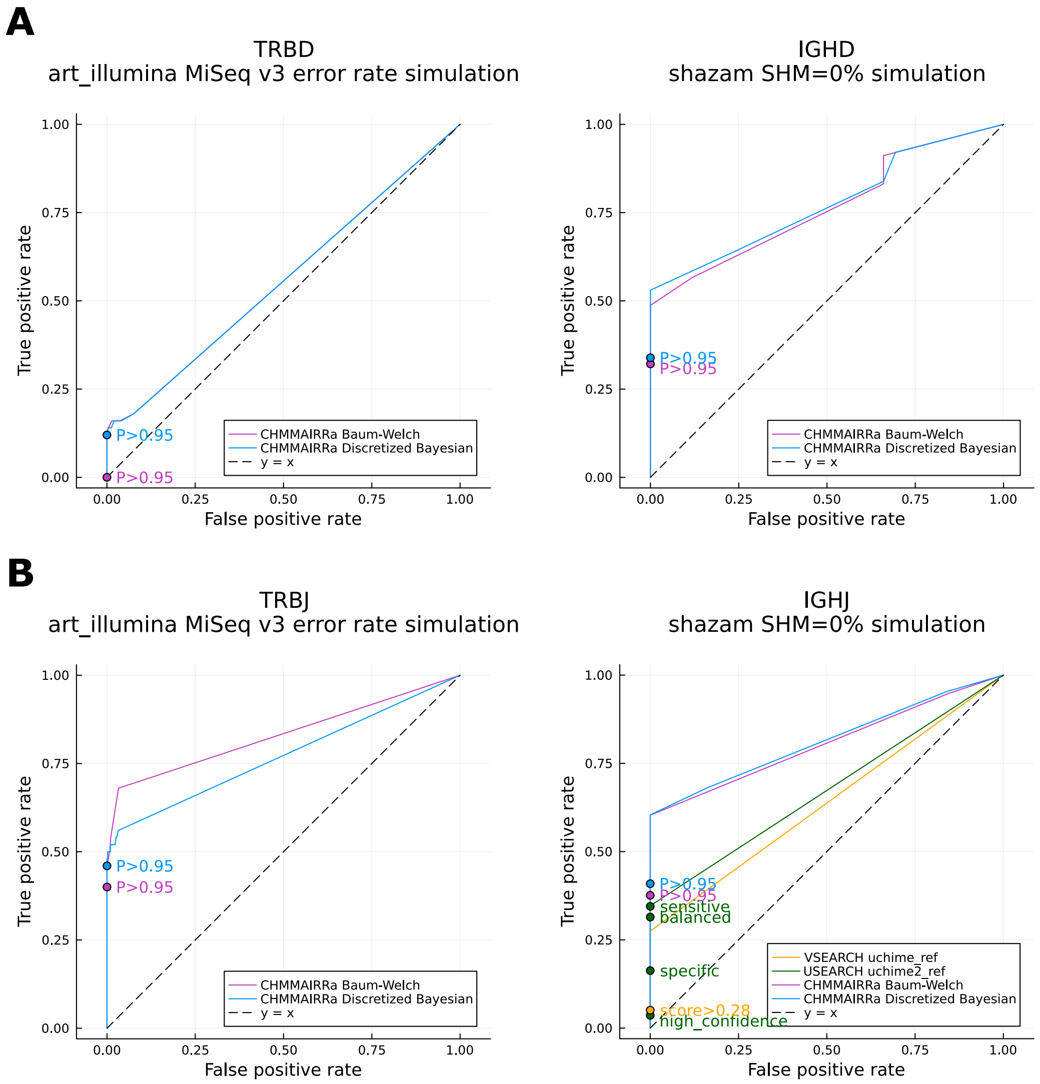

Supplementary Figure 1: CHMMAIRRa's accuracy on D and J gene simulations. ROCs for (A) TRBD and IGHD sequences and (B) TRBJ and IGHJ sequences. Errors in TRBs were simulated using ART, while IGH SHM was simulated by shazam's shmulateSeq. Labeled points indicate default thresholds for CHMMAIRRa (posterior probability > 0.95), VSEARCH (score > 0.28), and the four USEARCH modes representing varying levels of detection sensitivity (high_confidence, specific, balanced, and sensitive). Colors match the corresponding ROC curves. Methods achieving zero true positives on specific datasets are excluded from those panels

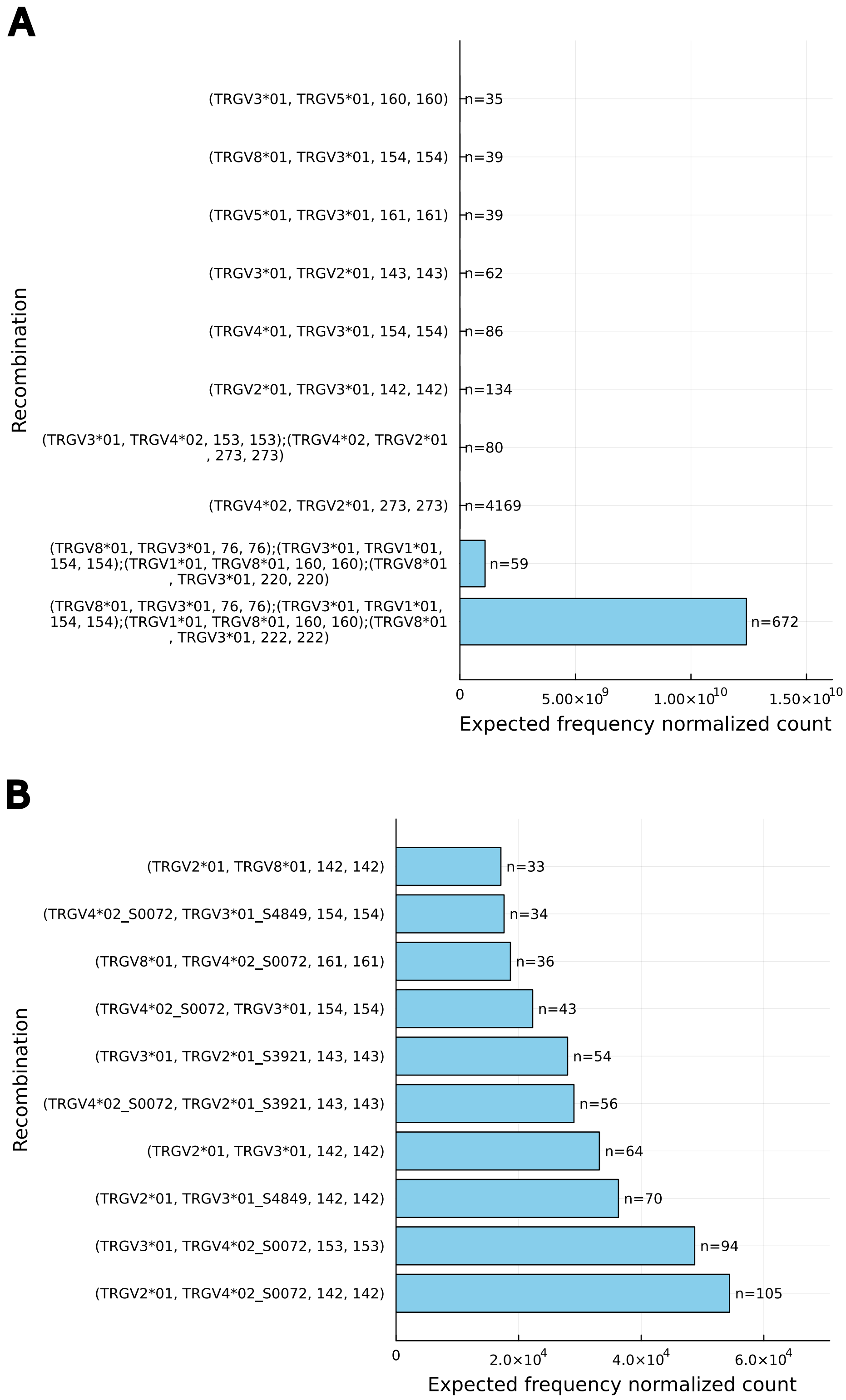

Supplementary Figure 2: Normalized counts of recombinations detected by CHMMAIRRa in the TRG/D04 library (Corcoran et al.) analyzed with the (A) the IMGT database and (B) the donor's personalized genotype. Normalization was performed by dividing the recombination counts by the corresponding allele frequencies. The overrepresented recombination in (A) corresponds to the TRGV7*01 allele, an allele absent from the functional IMGT database.

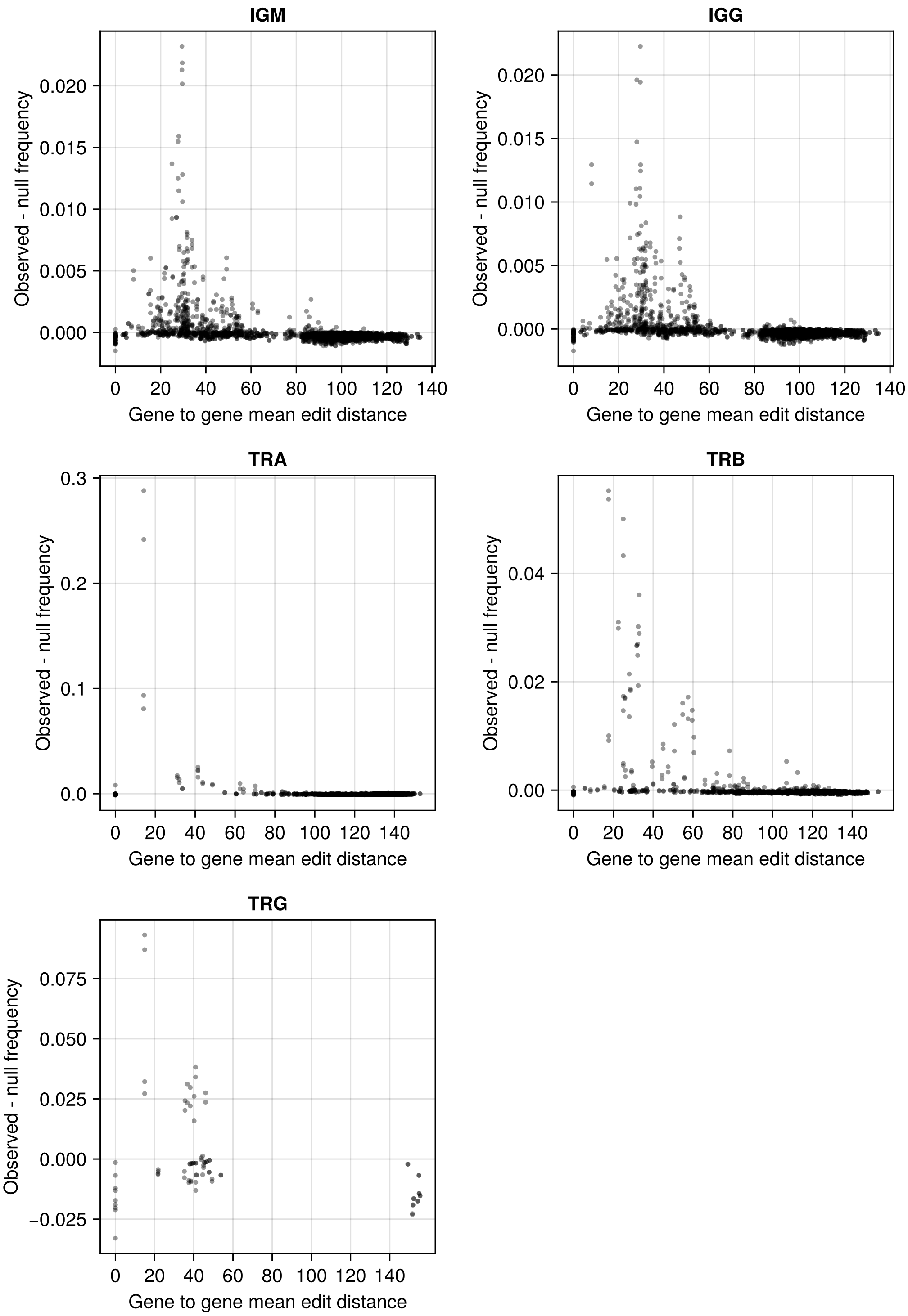

Supplementary Figure 3: Relationship between intergene edit distance and deviation from expected recombination frequency. Each dot represents recombinations involving two specific genes. Observed frequency is the average co-occurrence of genes across all datasets (excluding the PCR conditions dataset). Expected frequency is the product of the two genes' frequencies. Method settings listed in Supplementary Data 1 and datasets in the data availability section.

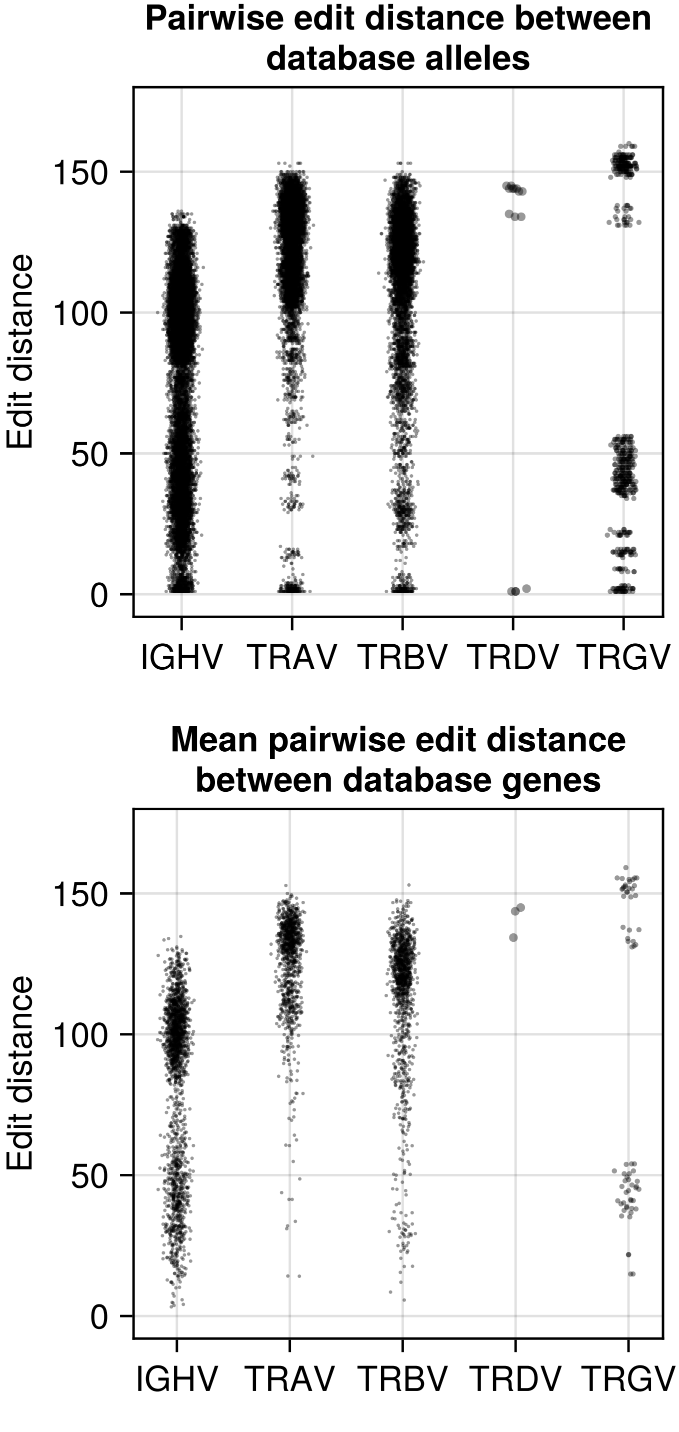

Supplementary Figure 4: Levenshtein distances from adaptive immune receptor databases: (A) between all pairs of V alleles and (B) averaged per-gene. The lack of closely related TRDV alleles may explain the scarcity of chimeras in TRD libraries. Database versions are provided in Supplementary Data 1.

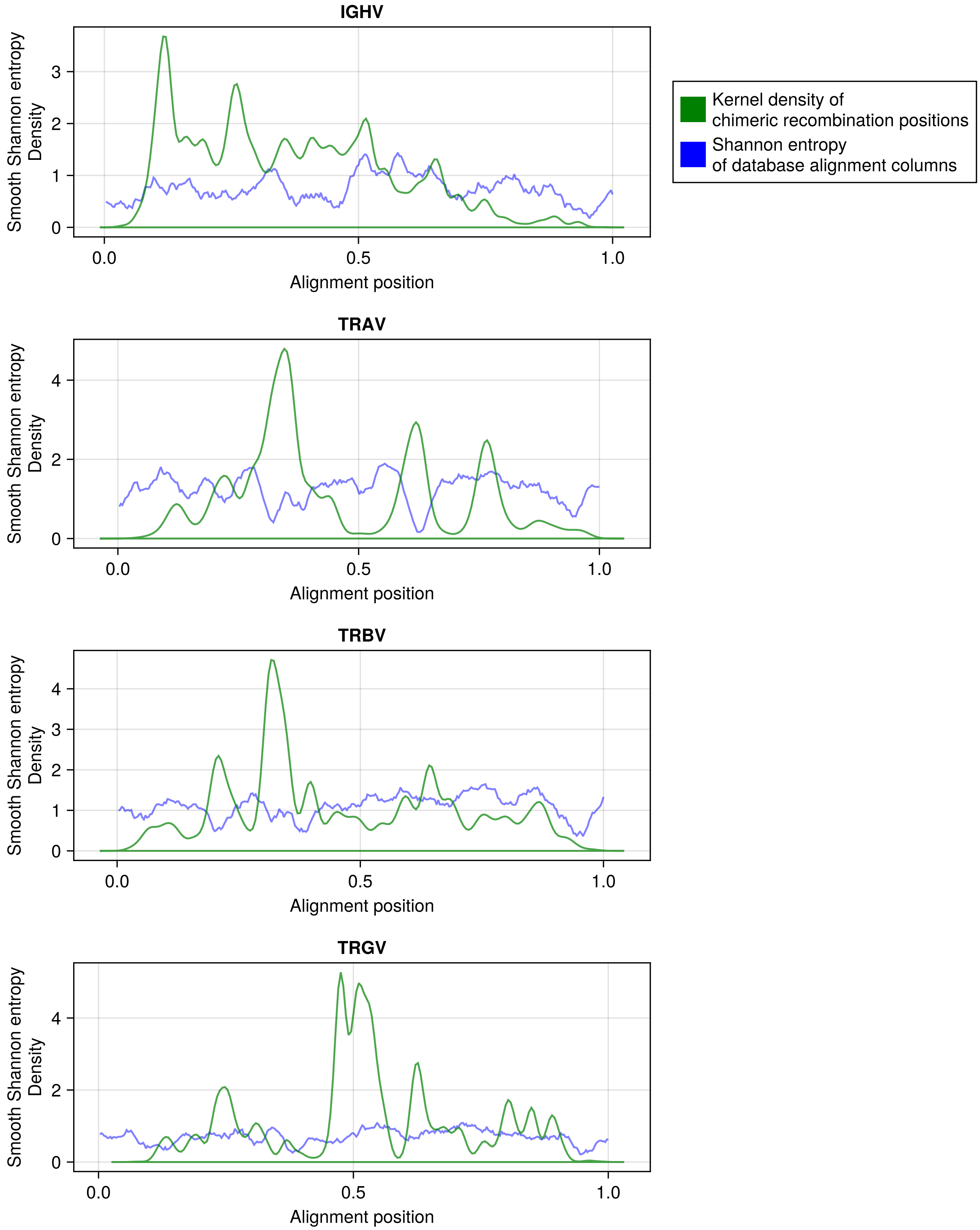

Supplementary Figure 5: Distributions of recombination positions across all real datasets (excluding the PCR conditions dataset). Kernel density estimates of recombination position (green) are overlaid on smoothed per-column Shannon entropies (blue) of database alignments. We hypothesized that conserved regions (lower entropy) recombine more readily, but no clear relationship between entropy and the recombination frequency was observed.

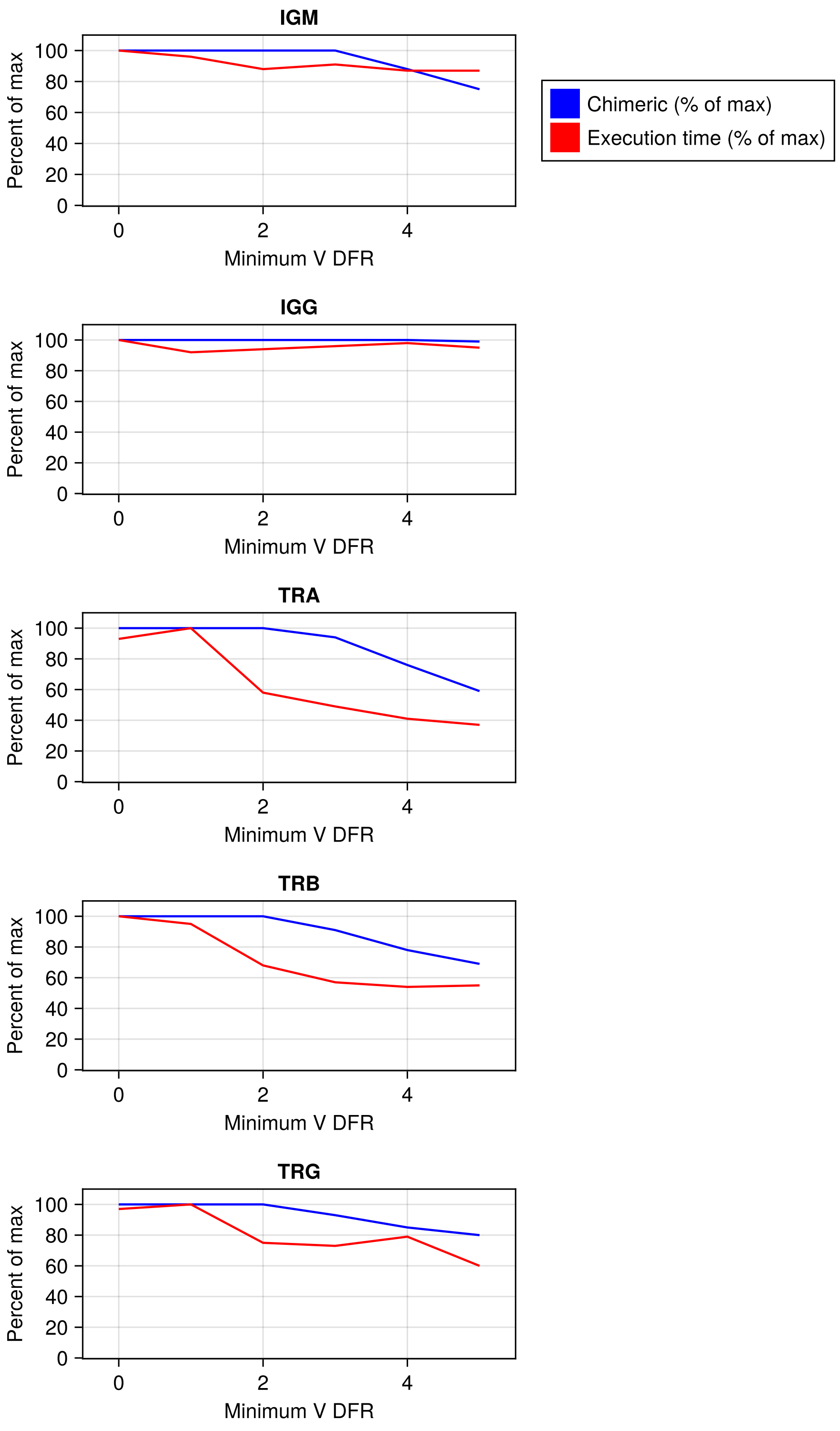

Supplementary Figure 6: CHMMAIRRa's chimerism detection rates and execution times as a percentage of their maximums across differences from reference (DFR) thresholds. DFR=1 indicates that sequences differing with <=1 difference from their closest reference are classified as non-chimeric. Because IgM and IgG sequences often carry SHM, increasing the threshold to DFR=5 provides little performance gain, whereas TCR data show a sharp reduction in run time. One library was analyzed per chain: IgM (IML3694_25cycle), IgG (IML3694_post-vax), TRA (GKH_TCR/TRA/D01), TRB (GKH_TCR/TRB/D01), and TRG (GKH_TCR/TRG/D01).

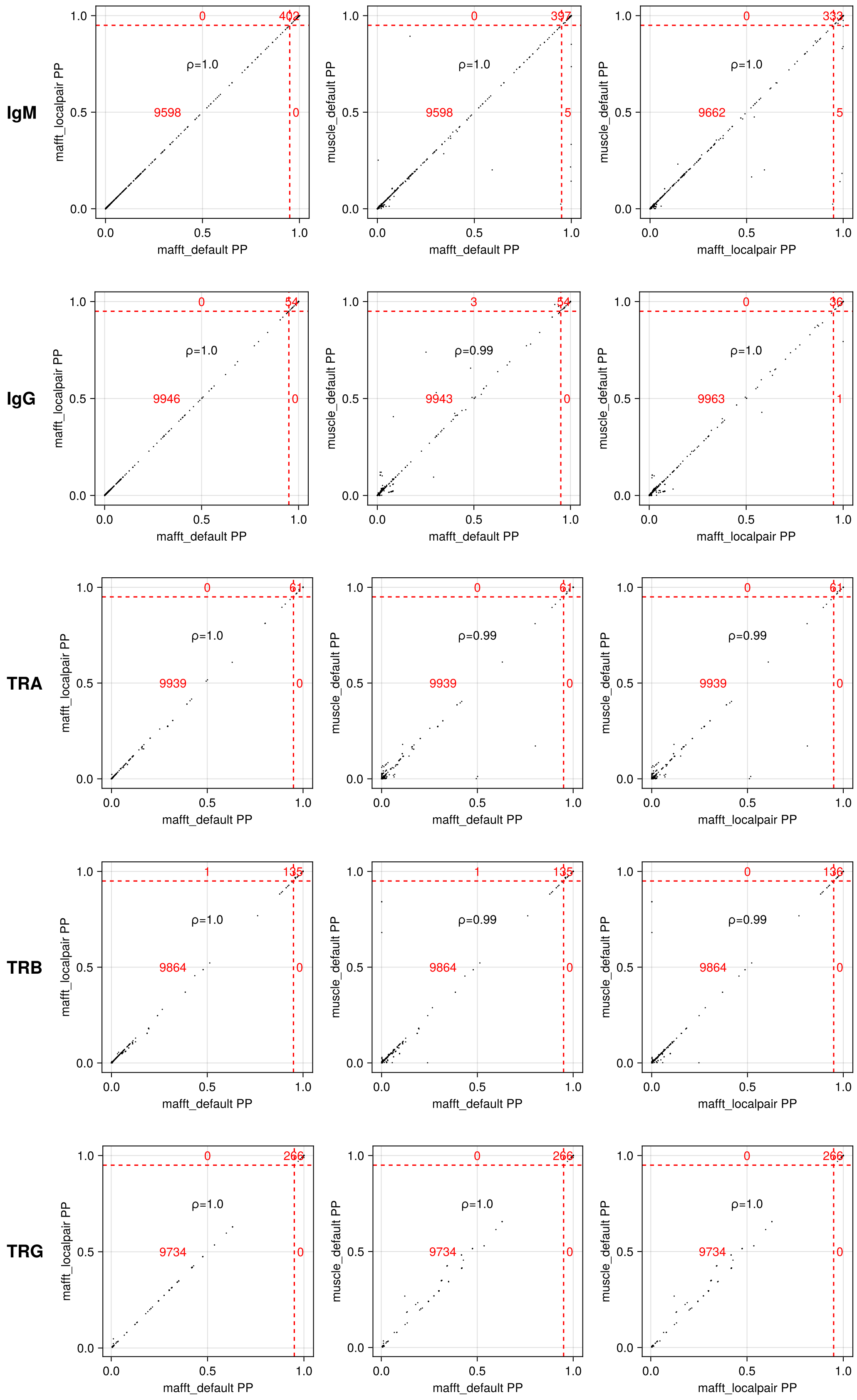

Supplementary Figure 7: Posterior probabilities of chimerism (PP) for V sequences of each chain across 3 alignment methods applied to the personalized genotype V databases. Alignment methods were MAFFT with –localpair option (mafft_localpair), MAFFT without options (mafft_default), and muscle without options (muscle_default). ρ indicates the Pearson correlation coefficient. One library was analyzed per chain: IgM (IML3694_25cycle), IgG (IML3694_post-vax), TRA (GKH_TCR/TRA/D01), TRB (GKH_TCR/TRB/D01), and TRG (GKH_TCR/TRG/D01).
